# Prognostic significance of microvascular invasion in tumor stage for hepatocellular carcinoma

**DOI:** 10.1186/s12957-017-1292-3

**Published:** 2017-12-19

**Authors:** Yong Keun Park, Sung Kyu Song, Bong-Wan Kim, Seung-Keun Park, Chul-Woon Chung, Hee-Jung Wang

**Affiliations:** 1 Department of Surgery, School of Medicine, Ajou University, 164 World cup-ro, Yeongtong-gu, Suwon, 16499 South Korea; 2 Department of Surgery, International St. Mary’s Hospital, Catholic Kwandong University, Incheon, South Korea; 3 Department of Supercomputing, M&S Technology Development, Korea Institute of Science and Technology Information, Daejeon, South Korea

**Keywords:** Alpha-fetoprotein, Hepatectomy, Vascular invasion, Tumor node metastasis stage, Tumor recurrence

## Abstract

**Background:**

The presence of microvascular invasion (McVI) in hepatocellular carcinoma (HCC) has been proposed as a cause of recurrence and poor survival, although this has not been officially emphasized in staging systems. Thus, we conducted a retrospective study to investigate the prognostic importance of McVI in tumor staging in patients with HCC who underwent hepatic resection.

**Methods:**

A retrospective analysis was performed of patients who underwent hepatic resection for HCC at our center from 1994 to 2012. Patients with HCC were classified into four groups based on the presence of McVI and extent of gross vascular invasion (VI).

**Results:**

The 5-year overall and recurrence-free survival rates of 676 patients were 63.3 and 42.6%, respectively. There was no difference in tumor recurrence or survival rate between patients with HCC and McVI without gross VI and those with gross VI confined to segmental/sectional branches. Multivariate analysis revealed that the extent of VI based on the presence of McVI and gross VI was independently associated with tumor recurrence and overall survival.

**Conclusions:**

McVI was revealed to be an important risk factor similar to gross VI confined to a segmental/sectional branch in patients with HCC who underwent hepatic resection. This finding should be considered when estimating the stage for prognosis.

## Background

Hepatocellular carcinoma (HCC) is one of the most commonly diagnosed cancers and is responsible for a high incidence of cancer-related deaths throughout the world. [1] However, treatment with curative intention, such as hepatic resection, liver transplantation (LT), and locoregional therapies, can only be applied in approximately 30% of patients with early-stage HCC [2]. Although these therapeutic modalities have improved the overall survival (OS), long-term outcomes remain poor because of high rates of tumor recurrence. Vascular invasion (VI) is a key contributor to tumor recurrence, which leads to dismal outcomes in patients with HCC [3].

When HCC tumor progresses, it may invade neighboring vessels [4]. VI by tumor cells is a well-recognized negative prognostic feature of HCC, which has been reflected in official staging systems [5–7]. In the tumor node metastasis (TNM) stage based on the criteria of the Liver Cancer Study Group of Japan (LCSGJ), VI is one of three factors for determining the T stage with tumor size and numbers [6]. According to the Barcelona Clinic for Liver Cancer (BCLC) staging, HCC with gross VI is classified as advanced stage, which most likely will not benefit from curative treatment [7]. However, it remains unclear how much microvascular invasion (McVI) provides prognostic information for patients with HCC from the viewpoint of the extent of tumor invasion or extension.

Studies evaluating patients stratified by various predictors of recurrence risk have identified McVI as a factor that can affect the prognosis of postoperative recurrence [8, 9]. McVI is also reported in several studies to be an important risk factor for HCC recurrence after LT [10, 11]. Subsequent studies have focused on preoperative prediction of McVI to aid the decision-making process for optimal treatment option in patients with HCC [12, 13].

Considered as the first step of metastatic dissemination via the vascular route, prognostic impact of McVI may be intuitively thought to be placed between non-VI and gross invasion of vessels. However, there is no strong evidence to support this speculation. The protocol developed by the College of American Pathologists considers McVI the same as gross VI confined to segmental/sectional branches of HCC on the current American Joint Committee on Cancer (AJCC)/International Union for Cancer Control (UICC) tumor TNM staging system [14]. Unfortunately, there is no mention of the prognostic significance of McVI on other staging systems, such as LCSGJ TNM or BCLC [6, 7]. In this retrospective study, we aimed to clarify the importance of McVI as the degree of local tumor invasion or extension in tumor stage for HCC.

## Methods

A retrospective analysis was performed on a database of patients who underwent surgical procedures for HCC at our center between September 1994 and December 2012. Data were extracted from prospectively collected database records, which included demographics, etiology of underlying liver disease, pathological findings of the specimen, surgical results, and oncological outcomes. Patients lost during follow-up were censored.

We preferentially considered and attempted surgical resection for all patients newly diagnosed with HCC in the Department of Surgery and all referred patients from the Department of Gastroenterology and other institutions if liver function was preserved and the state of HCC was not technically inoperable. Liver function was assessed by the Child–Turcotte–Pugh (CTP) classification and indocyanine green retention rate at 15 min (ICG-R15) value. For many years, our approach to determine the extent of resection has been based on a prediction scoring system [15]. We did not abandon hepatic resection because of the existence or extent of gross VI. Major hepatic resection was defined as the removal of three or more segments according to the Brisbane classification [16]. Intraoperative ultrasound was routinely used to detect any additional nodules and to aid in the determination of the most optimal resection plane.

Tumors were staged based on postoperative pathological findings according to the AJCC/UICC TNM and LCSGJ staging system [5, 6]. McVI was defined by a tumor within a vascular space lined by endothelium, identified only on microscopy in the capsule or noncapsular fibrous septa or liver tissue surrounding the tumor [17]. In all cases, tumor grade was defined by the poorest degree of differentiation using the Edmondson–Steiner grades, identified within the tumor upon pathological analysis of the entire specimen [18]. Portal vein tumor thrombus (PVTT) and hepatic vein tumor thrombus (HVTT) was classified into five and four groups, respectively, according to the General Rules for the Study of Primary Liver Cancer by the Korean Liver Cancer Study Group [19].

Follow-up investigations consisted of imaging studies with serum α-fetoprotein (AFP) level. Biochemical liver function tests, AFP level, and abdominal computed tomography (CT) scan were conducted every 3 months after discharge during the first 2 years and approximately every 3–6 months for the following years. Tumor recurrence was diagnosed by the combination of elevated tumor markers and consistent radiological findings. If recurrence was highly suspected without clear evidence on an imaging study, hepatic arteriography and lipiodol CT scans were performed. Patients with tumor recurrence were managed with various therapeutic modalities, including local ablation, re-resection, and salvage LT. Patients with multiple or large tumors and/or hepatic dysfunction underwent transcatheter arterial chemoembolization (TACE). Targeted therapy with sorafenib and radiation therapy were also adopted for advanced or metastatic tumors.

### Statistical analysis

Variables preoperatively and pathologically stratified were analyzed using univariate and multivariate analyses to determine independent predictors of oncological outcome. All continuous variables were expressed as mean ± standard deviation or median (minimum–maximum range). The optimal cutoff values for continuous variables for use in the Kaplan–Meier survival analyses were estimated by receiver operating characteristic (ROC) curve analysis. Survival rates and curves were estimated using the Kaplan–Meier method and compared using the log rank test. Multivariate analysis was performed using the Cox regression proportional hazards model to identify independent factors that determined recurrence-free survival (RFS) and OS. All statistical analyses were performed using *R*-packages, version 3.3.1 [20]. All *P* values < 0.05 were considered statistically significant.

## Results

**Fig. 1:**
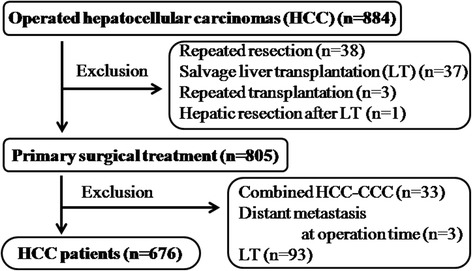
Flow diagram shows the selection of patients who were eligible for this study. CCC cholangiocarcinoma

A total of 884 surgical procedures for HCC were performed during the study period. Patients who underwent primary hepatic resection were eligible for the study. Exclusion criteria were patients who underwent primary LT for HCC (*n* = 93) and those undergoing reoperation (*n* = 79), such as repeated hepatic resection, salvage or repeated LT, and hepatic resection following LT. The diagnosis of HCC was confirmed by pathological examination in all cases. A total of 33 patients who had combined HCC and cholangiocarcinoma and three with distant metastasis at the operation time were also excluded. This retrospective study was performed on the remaining 676 patients (Fig. 1), and their clinicopathological details are summarized in Table 1. There were 530 male (78.4%) and 146 female (21.6%) patients (median age, 52 years; range, 20–76 years). Among the patients, 516 (78.5%) tested positive for serum hepatitis B surface antigen and 38 (6%) tested positive for hepatitis C antibody; 36 (5.3%) and 14 (2.1%) patients had CTP classes B and C, respectively, and 623 (92.6%) had class A disease. Serum AFP level was normal in 197 patients (29.6%), abnormal but less than 400 ng/mL in 251 (37.7%), and more than 1000 ng/mL in 217 (32.7%). Two hundred thirty-two (40.0%) patients underwent preoperative TACE. Among the operations, 262 (38.7%; approximately 4/10 rate) were major hepatic resections, whereas 184 (27.2%) were segmentectomies or bisegmentectomies and 230 (34.1%) were minor resections.

**Table 1:** Comparison of clinicopathological data for patients classified into four groups

	Total (*n* = 676)	Group A (*N* = 335)	Group B (*N* = 193)	Group C (*N* = 103)	Group D (*N* = 45)	*P* value
Gender						0.28
Male	530 (78.4%)	260 (77.6%)	146 (75.6%)	85 (82.5%)	39 (86.7%)	
Female	146 (21.6%)	75 (22.4%)	47 (24.4%)	18 (17.5%)	6 (13.3%)	
Ages (years)	52.3 ± 10.2	52.7 ± 10.0	52.1 ± 10.7	51.4 ± 10.5	51.7 ± 9.4	0.244
Hepatitis B surface antigen						0.171
Negative	141 (21.5%)	76 (23.2%)	43 (23.2%)	17 (17.0%)	5 (11.1%)	
Positive	516 (78.5%)	251 (76.8%)	142 (76.8%)	83 (83.0%)	40 (88.9%)	
Hepatitis C antibody						0.351
Negative	593 (94.0%)	293 (93.3%)	166 (93.3%)	91 (94.8%)	43 (100.0%)	
Positive	38 (6.0%)	21 (6.7%)	12 (6.7%)	5 (5.2%)	0 (0.0%)	
Platelet count (× 1000/uL)	170 ± 81	157 ± 75	174 ± 82	187 ± 85	204 ± 96	< 0.001
Serum creatinine (mg/dL)	1.0 ± 0.9	1.0 ± 0.8	1.1 ± 1.3	0.9 ± 0.2	0.9 ± 0.2	0.49
Serum albumin (g/dL)	4.0 ± 0.5	4.0 ± 0.5	3.9 ± 0.5	4.0 ± 0.4	3.8 ± 0.5	0.368
Serum total bilirubin (mg/dL)	0.9 ± 1.2	0.9 ± 0.6	1.1 ± 1.9	0.9 ± 1.0	0.9 ± 0.5	0.587
Serum AST (U/L)	59.5 ± 66.4	50.9 ± 40.5	69.6 ± 102.9	57.3 ± 37.0	85.1 ± 65.2	0.002
Serum ALT (U/L)	56.1 ± 59.8	54.2 ± 48.3	59.9 ± 84.0	50.7 ± 38.2	66.7 ± 52.8	0.471
Prothrombin time (seconds)	12.4 ± 1.4	12.5 ± 1.4	12.5 ± 1.6	12.1 ± 1.3	12.8 ± 1.4	0.587
ICG-R15 (%)	14.9 ± 9.8	15.4 ± 9.9	13.7 ± 7.9	15.8 ± 13.2	14.3 ± 8.5	0.608
Child–Turcotte–Pugh classification						0.062
A	623 (92.6%)	317 (95.2%)	170 (88.5%)	94 (91.3%)	42 (93.3%)	
B	36 (5.3%)	9 (2.7%)	16 (8.3%)	8 (7.8%)	3 (6.7%)	
C	14 (2.1%)	7 (2.1%)	6 (3.1%)	1 (1.0%)	0 (0.0%)	
Alpha-fetoprotein (ng/mL)	3705.9 ± 10,891.1	913.7 ± 4020.0	4598.0 ± 11,355.1	7336.6 ± 16,371.8	12,966.7 ± 18,814.6	< 0.001
Preoperative TACE						0.086
No	413 (64.0%)	195 (60.7%)	126 (69.6%)	68 (68.7%)	24 (54.5%)	
Yes	232 (40.0%)	126 (39.3%)	55 (30.4%)	31 (31.3%)	20 (45.5%)	
Types of hepatic resection						< 0.001
Major	262 (38.7%)	87 (26.0%)	77 (39.9%)	57 (55.4%)	41 (91.1%)	
Sectionectomy	184 (27.2%)	104 (31.0%)	45 (23.3%)	33 (31.7%)	2 (4.4%)	
Segmentectomy or less	230 (34.1%)	144 (43.0%)	71 (36.8%)	13 (12.9%)	2 (4.4%)	
Size of the tumor (cm)	5.4 ± 3.9	3.9 ± 2.9	6.1 ± 4.2	7.2 ± 3.7	9.5 ± 4.2	< 0.001
Tumor number						0.001
Single	536 (79.3%)	287 (85.7%)	142 (73.6%)	76 (73.8%)	31 (68.9%)	
Multiple	140 (20.7%)	48 (14.3%)	51 (26.4%)	27 (26.2%)	14 (31.1%)	
Portal vein invasion						< 0.001
Negative	537 (79.4%)	335 (100.0%)	193 (100.0%)	7 (6.8%)	2 (4.4%)	
Positive	139 (20.6%)	0 (0.0%)	0 (0.0%)	96 (93.2%)	43 (95.6%)	
Hepatic vein invasion						< 0.001
Negative	647 (95.7%)	335 (100.0%)	193 (100.0%)	89 (86.4%)	30 (66.7%)	
Positive	29 (4.3%)	0 (0.0%)	0 (0.0%)	14 (13.6%)	15 (33.3%)	
Microvascular invasion						< 0.001
Negative	348 (51.5%)	335 (100.0%)	0 (0.0%)	9 (6.9%)	4 (4.7%)	
Positive	328 (48.5%)	0 (0.0%)	193 (100.0%)	94 (93.1%)	41 (95.3%)	
Intrahepatic metastasis						< 0.001
Negative	452 (66.9%)	265 (79.1%)	124 (64.2%)	48 (46.6%)	15 (33.3%)	
Positive	224 (33.1%)	70 (20.9%)	69 (35.8%)	55 (53.4%)	30 (66.7%)	
Histologic grading by Edmondson and Steiner’s classification						< 0.001
Negative	379 (60.7%)	227 (78.3%)	88 (46.1%)	46 (46.0%)	18 (41.9%)	
Positive	245 (39.3%)	63 (21.7%)	103 (53.9%)	54 (54.0%)	25 (58.1%)	
Microscopic resection margin						< 0.001
Negative	589 (87.9%)	312 (93.7%)	170 (89.0%)	79 (77.5%)	28 (63.6%)	
Positive	81 (12.1%)	21 (6.3%)	21 (11.0%)	23 (22.5%)	16 (36.4%)	
Cirrhosis						0.346
Negative	296 (47.0%)	141 (44.8%)	93 (50.8%)	47 (50.5%)	15 (38.5%)	
Positive	334 (53.0%)	174 (55.2%)	90 (49.2%)	46 (49.5%)	24 (61.5%)	
AJCC TNM stage						< 0.001
I	260 (38.4%)	265 (79.1%)	2 (1.0%)	0 (0.0%)	0 (0.0%)	
II	244 (36.1%)	40 (11.9%)	142 (73.6%)	65 (63.1%)	0 (0.0%)	
III-A	62 (9.2%)	14 (4.2%)	26 (13.5%)	24 (23.3%)	0 (0.0%)	
III-B	50 (7.4%)	0 (0.0%)	0 (0.0%)	0 (0.0%)	38 (84.4%)	
III-C	54 (8.0%)	15 (4.5%)	21 (10.9%)	12 (11.7%)	6 (13.3%)	
IV-A	6 (0.9%)	1 (0.3%)	2 (1.0%)	2 (1.9%)	1 (2.2%)	
LCSGJ TNM stage						< 0.001
I	78 (11.8%)	67 (20.6%)	11 (5.7%)	0 (0.0%)	0 (0.0%)	
II	329 (49.8%)	207 (63.7%)	114 (59.1%)	5 (5.1%)	3 (6.7%)	
III	183 (27.7%)	51 (15.7%)	63 (32.6%)	54 (55.1%)	15 (33.3%)	
IV	71 (10.7%)	0 (0.0%)	5 (2.6%)	39 (39.8%)	27 (60.0%)	

*Group A* no microvascular invasion (McVI) or gross vascular invasion (VI), *Group B* McVI without gross VI, *Group C* VI confined to segmental/sectional branches, *Group D* gross VI within/beyond major vascular branches, *ICG-R15* indocyanine green retention rate at 15 min, *TACE* transcatheter arterial chemoembolization, *AJCC TNM* American Joint Committee on Cancer Tumor Node Metastasis, *LCSGJ* the Liver Cancer Study Group of Japan

Overall median follow-up period was 40 (1–204) months. The 90-day mortality rate after hepatic resection because of post-hepatectomy liver failure or sepsis was 1.9% (13 of 676). During the follow-up period, 55.1% (365 of 663) of the patients had tumor recurrence and 35.6% (236 of 663) died. The 5-year OS and RFS rates were 63.3 and 42.6%, respectively.

Pathological analysis postoperatively revealed that 328 patients (48.5%) had combined McVI: 193 had McVI without and 135 had McVI with gross VI. According to the extent of PVTT, 537 patients (79.4%) had Vp0, 58 (8.6%) had Vp1, 41 (6.1%) had Vp2, 22 (3.3%) had Vp3, and 18 (2.7%) had Vp4. A total of 29 patients (4.3%) had tumor with hepatic vein invasion. Based on the extent of HVTT, the patients were classified into four groups: Vv0 (*n* = 647), Vv1 (*n* = 19), Vv2 (*n* = 5), and Vv3 (*n* = 5). The patients were also divided into four groups based on the existence of McVI and extent of gross VI: group A, no McVI or gross VI; group B, McVI without gross VI; group C, VI confined to segmental/sectional branches (Vp1–2 or Vv1); and group D, gross VI within/beyond major vascular branches (Vp3–4 or Vv2–3). The relationship between groups A–D and the clinicopathological factors are shown in Table 1.

**Fig. 2:**
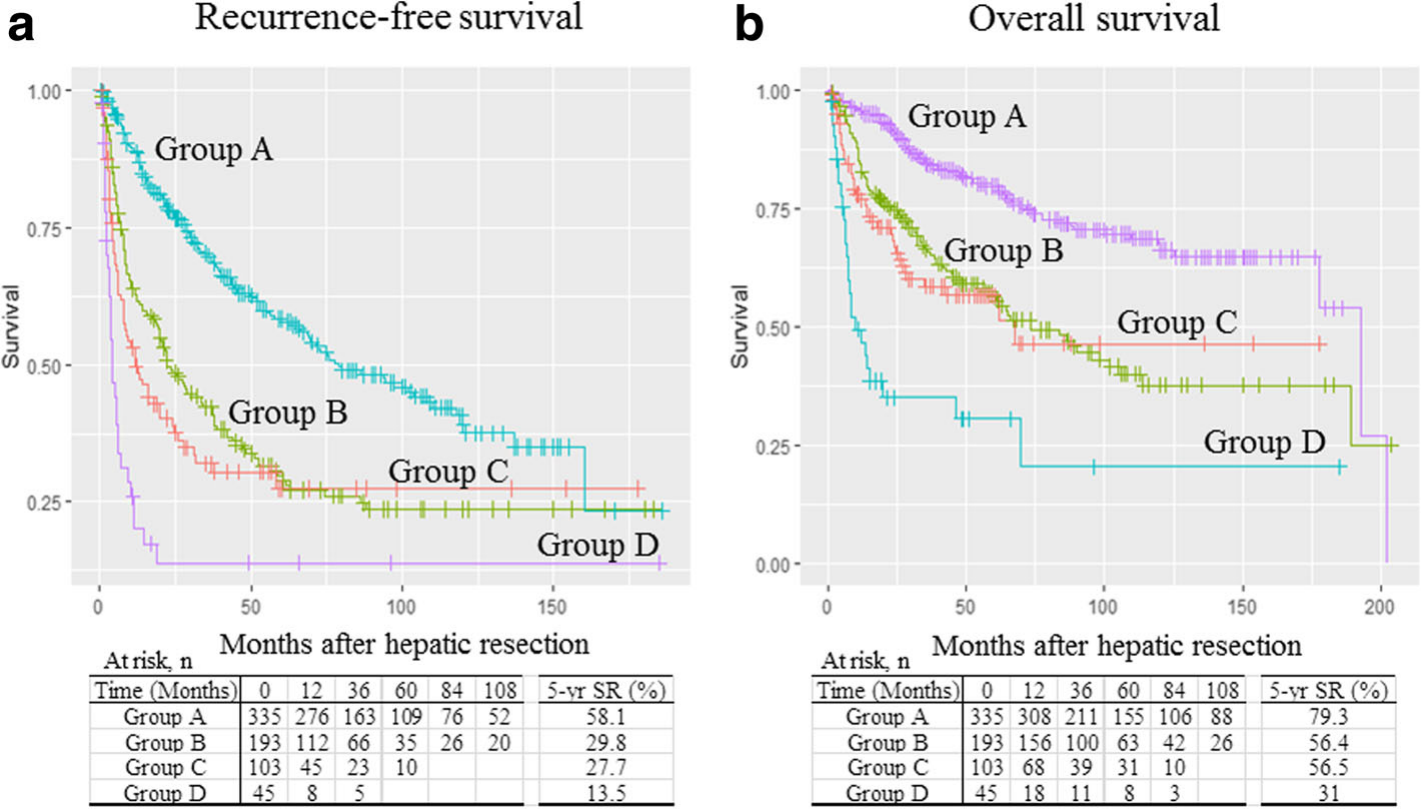
Comparison of **a** recurrence-free and **b** overall survival of patients stratified into groups A–D. No significant changes are seen between groups B and C (recurrence-free survival, *P* = 0.18; overall survival, *P* = 0.43). SR survival rate

We observed marked differences in groups of patients according to preoperative platelet count, aspartate aminotransferase (AST) and AFP level, and tumor size. There were also significant differences in the percentage of major operations, multiplicity of tumors, intrahepatic metastasis, tumor histology, histological involvement of the resection margin, and tumor stage. Group D had the highest preoperative platelet counts, AST and AFP levels, and tumor sizes. In comparison, group A had the lowest values of these parameters. In addition, group D had the highest rate of major hepatic resection (91.1%), followed by group C (55.4%), whereas group A had the lowest rate of major hepatic resection (26.0%). We observed that 31.1% of patients in group D had multiple tumors, whereas 85.7% in group A had a single tumor. Moreover, group D had the highest proportions of intrahepatic metastasis and worse tumor histological grade (66.7 and 58.1%, respectively), followed by group C (53.4 and 54.0%, respectively). In addition, group D had the highest rate of positive surgical margins, followed by group C (36.4 and 22.5%, respectively), whereas group A had the lowest rate of intrahepatic metastasis (20.9%), worse tumor histological grade (21.7%), and positive surgical margins (6.3%). When comparing the survival curves according to these four groups, group D demonstrated significantly worse survival compared to the other groups: RFS and OS for groups A versus D (*P* < 0.001), B versus D (*P* < 0.001), and C versus D (*P* = 0.001). Moreover, groups B and C showed markedly worse outcomes than group A: RFS and OS for groups A versus B (*P* < 0.001) and A versus C (*P* < 0.001). However, no significant differences in RFS and OS were noted between groups B and C: 5-year RFS rates, 29.8 and 27.7%, respectively (*P* = 0.18); 5-year OS rates, 56.4 and 56.5%, respectively (*P* = 0.43; Fig. 2). Therefore, patients were reclassified into three groups (groups A vs. B/C vs. D) for further analysis in a multivariate model.

**Fig. 3:**
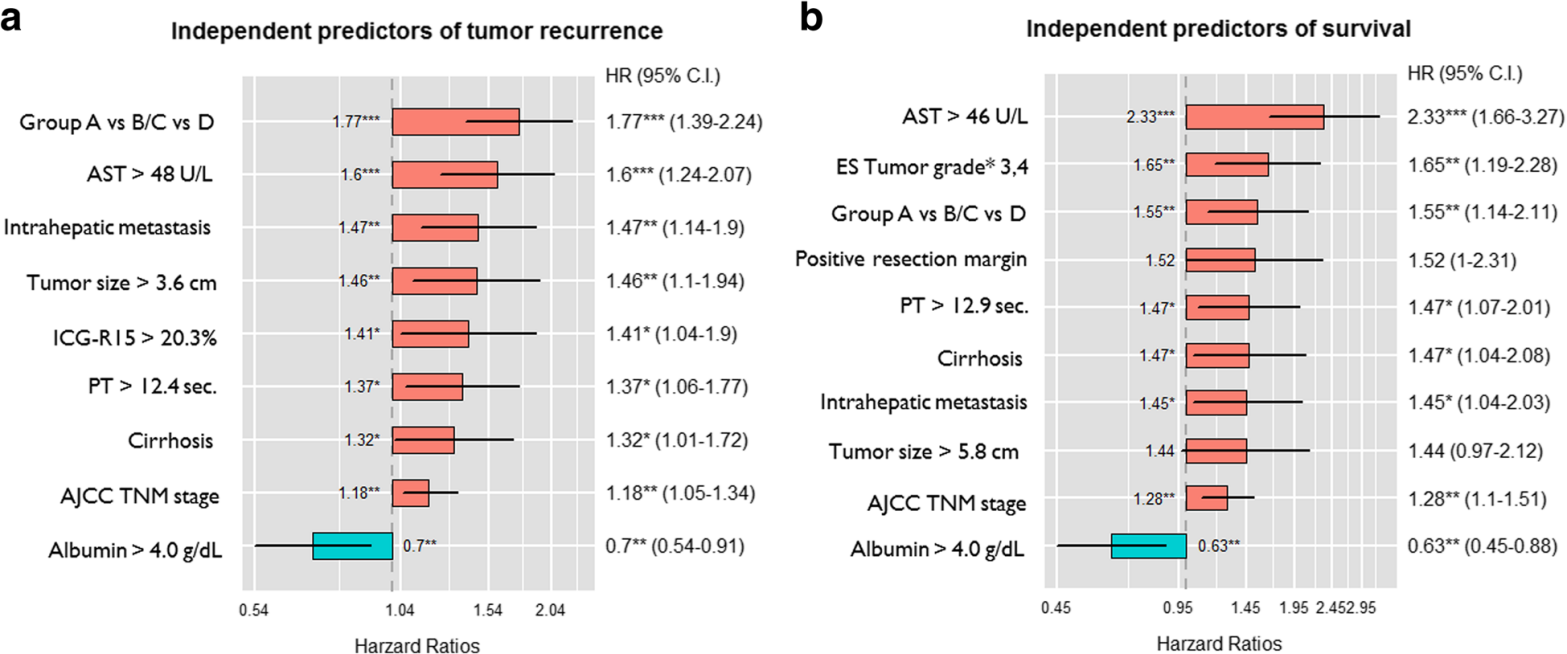
Summary of statistically significant clinicopathological factors on **a** recurrence-free survival and **b** overall survival using the Cox regression proportional hazards model. AJCC TNM American Joint Committee on Cancer Tumor Node Metastasis, AST aspartate aminotransferase, ES Edmondson–Steiner classification, HR hazard ratio, C.I. confidence interval, ICG-R15 indocyanine green retention rate at 15 min, PT prothrombin time

Univariate analysis according to clinicopathological factors was used to find predictors of tumor recurrence and survival. Cutoff values for the continuous variables (preoperative platelet count, AST and AFP levels, etc.) were calculated by ROC curve analysis (Table 2). Multivariate analysis revealed predictors that were independently associated with tumor recurrence and OS. The extent of VI (groups A vs. B/C vs. D), higher AST level, existence of intrahepatic metastasis, larger tumor size, elevated ICG-R15 value, prolonged prothrombin time, liver cirrhosis, and advanced tumor stage were independent risk factors for tumor recurrence. Albumin level over 4 g/dL was a positive risk factor for prognosis (Fig. 3a). Among the abovementioned risk factors, larger tumor size and elevated ICG-R15 value were not significantly related to poor OS. Worse histological grade and positive surgical margins were independent predictive factors of worse survival (Fig. 3b).

**Table 2:** Univariate analysis of factors predictive of recurrence-free and overall survival

Factors	No. of patients	MDFST^a^ (95% CI)	*P* value	Factors	No. of patients	MOST^b^ (95% CI)	*P* value
Gender			0.602	Gender			0.431
Male	515	42.0 (33.4–53.0)		Male	520	178.0 (98.0–NA)	
Female	144	34.0 (26.1–71.5)		Female	145	189.0 (67.0–NA)	
Ages (years)			0.167	Ages (years)			0.428
< 51	274	50.5 (32.4–72.2)		< 42	90	189.0 (64.5–NA)	
≥ 51	385	37.5 (28.8–45.6)		≥ 42	575	178.0 (106.0–NA)	
Hepatitis B or C infection status			0.722	Hepatitis B or C infection status			0.964
Negative	109	53.0 (37.5–NA)		Negative	110	193.0 (NA–NA)	
Positive	537	38.0 (30.9–49.7)		Positive	541	120.0 (89.0–NA)	
Platelet count (× 1000/uL)			0.49	Platelet count (× 1000/uL)			0.026
≥ 200	165	38.0 (21.6–58.0)		≥ 294	43	77.5 (29.4–NA)	
< 200	483	39.1 (33.0–53.0)		< 294	611	178.0 (109.7–NA)	
Serum creatinine (mg/dL)			0.416	Serum creatinine (mg/dL)			0.55
< 1.2	538	43.4 (34.0–57.9)		< 1.2	542	178.0 (125.3–NA)	
≥ 1.2	60	37.5 (20.9–NA)		≥ 1.2	61	69.5 (61.2–NA)	
Serum albumin (g/dL)			< 0.001	Serum albumin (g/dL)			< 0.001
< 4.0	297	21.6 (16.0–27.8)		< 4.0	300	64.2 (49.0–89.0)	
≥ 4.0	361	69.0 (55.4–102.0)		≥ 4.0	364	189.0 (178.0–NA)	
Serum total bilirubin (mg/dL)			0.12	Serum total bilirubin (mg/dL)			0.049
< 1.5	592	40.0 (32.4–53.0)		< 0.8	352	193.0 (102.0–NA)	
≥ 1.5	55	28.0 (10.5–69.0)		≥ 0.8	301	120.0 (83.8–NA)	
Serum AST (U/L)			< 0.001	Serum AST (U/L)			< 0.001
< 48	397	69.7 (53.0–104.0)		< 46	375	193.0 (193.0–NA)	
≥ 48	261	20.0 (15.0–28.0)		≥ 46	289	59.1 (45.9–67.0)	
Serum ALT (U/L)			< 0.001	Serum ALT (U/L)			< 0.001
< 44	361	58.0 (43.9–86.1)		< 48	407	189.0 (178.0–NA)	
≥ 44	297	29.0 (21.6–38.6)		≥ 48	257	67.0 (56.2–110.0)	
Prothrombin time (seconds)			< 0.001	Prothrombin time (seconds)			< 0.001
< 12.4	321	63.0 (43.4–118.0)		< 12.9	392	193.0 (NA–NA)	
≥ 12.4	327	31.4 (23.2–39.0)		≥ 12.9	262	88.0 (64.5–NA)	
Indocyanine green retention rate at 15 min			0.001	Indocyanine green retention rate at 15 min			0.852
< 20.3	525	47.4 (37.5–63.0)		< 12.9	290	178.0 (89.8–NA)	
≥ 20.3	112	24.5 (18.0–36.7)		≥ 12.9	352	120.0 (84.0–NA)	
Child–Turcotte–Pugh classification			< 0.001	Child–Turcotte–Pugh classification			< 0.001
A	606	45.5 (37–58.6)		A	612	178 (125.3–NA)	
B or C	50	11.9 (6.0–27.7)		B or C	50	14 (9.7–40.2)	
Alpha-fetoprotein (ng/mL)			< 0.001	Alpha-fetoprotein (ng/mL)			< 0.001
< 12.6	223	53.0 (40.3–109.0)		< 16.8	261	193.0 (193.0–NA)	
≥ 12.6	426	30.2 (24.5–44.7)		≥ 16.8	394	98.0 (67.2–NA)	
Preoperative TACE			0.100	Preoperative TACE			0.017
No	396	51.0 (35.8–79.0)		No	403	178 (178.0–NA)	
Yes	232	37.5 (26.2–48.3)		Yes	231	120 (67.2–NA)	
Extent of resection			0.035	Extent of resection			0.0052
Major	255	31.4 (20.0–47.4)		Major	257	178 (69.5–NA)	
Minor	404	45.5 (36.79–61.4)		Minor	408	125 (101.0–NA)	
Size of tumor (cm)			< 0.001	Size of tumor (cm)			< 0.001
< 3.6	282	66.3 (51.0–99.9)		< 5.8	447	189.0 (178.0–NA)	
≥ 3.6	377	21.5 (16.0–28.8)		≥ 5.8	218	46.3 (28.0–83.8)	
Tumor number			0.317	Tumor number			0.259
Single	520	50.5 (38.6–66.0)		Single	526	189.0 (189.0–NA)	
Multiple	139	11.5 (8.8–21.6)		Multiple	139	34.7 (24.4–56.2)	
Extent of vascular invasion			< 0.001	Extent of vascular invasion			<0.001
Group A	326	79.0 (66.3–110.0)		Group A	329	193.0 (178.0–NA)	
Group B	191	22.4 (19.3–37.2)		Group B	191	73.4 (59.1–114.0)	
Group C	98	12.7 (8.0–24.5)		Group C	101	67.2 (34.8–NA)	
Group D	44	4.1 (3.6–7.1)		Group D	44	12.0 (7.1–NA)	
Intrahepatic metastasis			0.001	Intrahepatic metastasis			0.049
Negative	443	58.0 (45.5–75.2)		Negative	447	189.0 (189.0–NA)	
Positive	216	14.1 (10.4–20.5)		Positive	218	43.0 (33.2–63.3)	
Histologic grading by Edmondson and Steiner’s classification			0.111	Histologic grading by Edmondson and Steiner’s classification			0.004
I~II	370	47.4 (37.6–68.9)		Negative	373	178.0 (125.3–NA)	
III~IV	238	21.9 (16.0–37.4)		Positive	241	71.4 (59.1–110.0)	
Microscopic resection margin			0.065	Microscopic resection margin			0.019
Negative	572	45.6 (38.0–60.4)		Negative	578	189.0 (119.8–NA)	
Positive	81	8.8 (6.0–19.3)		Positive	81	28.7 (13.2–NA)	
Cirrhosis			< 0.001	Cirrhosis			< 0.001
Negative	288	68.9 (43.4–110.0)		Negative	291	193.0 (193.0–NA)	
Positive	325	30.4 (22.2–39.9)		Positive	328	89.0 (67.0–NA)	
American Joint Committee on Cancer TNM stage			< 0.001	American Joint Committee on Cancer TNM stage			< 0.001
I	259	102.0 (72.2-NA)		I	262	202.0 (NA–NA)	
II	242	31.6 (24.1–47.4)		II	244	98.0 (67.2–NA)	
III-A	62	8.6 (5.8–21.6)		III-A	63	34.9 (25.6–89.8)	
III-B	37	4.1 (3.6–11.1)		III-B	37	9.63 (6.8–NA)	
III-C	53	8.0 (4.6–14.1)		III-C	53	18.4 (12.9–83.8)	
IV-A	6	9.3 (7.8–NA)		IV-A	6	16.0 (4.67–NA)	
LCSGJ TNM stage			< 0.001	LCSGJ TNM stage			< 0.001
I	76	66.0 (51.0–NA)		I	77	NA (NA–NA)	
II	324	58.6 (45.5–94.4)		II	325	202.0 (202.0–NA)	
III	177	20.0 (13.9–39.1)		III	179	64.5 (43.0–178.0)	
IV-A	69	5.6 (3.6–9.2)		IV-A	70	13.8 (9.0–27.9)	

*CI* confidence interval, *NA* not available, *TNM* tumor node metastasis, *LCSGJ* the Liver Cancer Study Group of Japan

^a^Median disease-free survival time (month)

^b^Median overall survival time (month)

## Discussion

Our study demonstrated the clinical significance of McVI in a manner that has not been used in previous similar studies. When the influence of McVI was analyzed, tumor recurrence and survival rates of patients with HCC and McVI without gross VI (group B) were not different from those of patients with gross VI confined to segmental/sectional braches (group C) after hepatic resection. To the best of our knowledge, no study has directly compared the relative importance of McVI and gross VI on tumor recurrence and long-term survival of patients with HCC undergoing hepatic resection.

Previous studies have shown that McVI is an important factor affecting the prognosis of patients with HCC, especially after hepatic resection or LT [8–11]. However, it is difficult to find studies dealing with the significance of McVI as the degree of local tumor invasion or extension, despite the instinctive guess that it might be an intermediate state of VI between nonvascular invasion and gross tumor invasion of neighboring segmental vessels. We generally assumed that the risk of tumor recurrence, as well as death, would be significantly lower in patients with HCC and McVI without gross VI (group B) than in those with gross VI confined to the segmental/sectional branch (group C). Contrary to our expectation, McVI has similar prognostic power compared with gross VI confined to the segmental/sectional branch (Fig. 2).

Despite its importance, official staging systems, such as the LCSGJ TNM, and BCLC staging systems, contain no mention of McVI [6, 7]. The protocol developed by the College of American Pathologists considers McVI the same as gross VI of HCC on the AJCC/UICC TNM staging system, although related studies are difficult to find [14]. Then, we focused on whether tumor stage would be influenced by McVI. Our primary goal for this study has been to evaluate the importance of McVI in tumor stage for HCC. In the present study, patients with HCC and McVI without gross VI (group B) had similar outcomes of tumor recurrence and survival compared with those with gross VI confined to segmental/sectional branches (group C). When compared with patients with gross VI within/beyond major vascular branches (group D), patients in group B/C had lower rates of tumor recurrence and good survival (Fig. 3). Our results tended to support the protocol of the College of American Pathologists.

While survival outcomes are notoriously worse in patients with gross VI than in those without gross VI (group A), those with HCC with gross VI confined to segmental/sectional branches (group C) had better outcomes than those with gross VI within/beyond major vascular branches (group D). Several studies have dealt with the extent of gross VI and its clinical impact on HCC [21, 22]. Survival outcomes of these previous studies are comparable to those of our study. The essential of cancer surgery is complete removal of tumor with free and safe margins. From the viewpoint of surgical principle, resection of a tumor with gross VI isolated within segmental/sectional branches could be considered as curative intention treatment through major hepatic resection without exposure of tumor thrombus margins on the portal or hepatic vein. However, resection of tumor with gross VI within/beyond major vascular branches should be considered as palliative treatment because exposure of tumor thrombus inside the vessels is not avoidable.

In the BCLC staging system, patients with HCC and VI (preoperative gross VI on image studies) are classified as having stage C disease and guided into treatments with palliative intent [7]. There is no mention of McVI because the BCLC system is designed to guide treatment according to preoperative patient information and McVI can be postoperatively confirmed through resected specimen. Therefore, there have been efforts to preoperatively detect McVI in HCC. Tools, such as performance of prothrombin induced by vitamin K absence-II and fluorodeoxyglucose-positron emission tomography, have already been suggested to preoperatively predict McVI of HCC [23, 24]. Moreover, several studies have been conducted using radiological imaging, molecules or gene expression from tumor, and other preoperative tumor characteristics [25–27]. At this point, a practical question can be raised. Should patients with HCC be guided to palliative treatment if McVI can be preoperatively determined? We suggest that treatments with curative intent should be recommended for patients with HCC if they have good liver function, based on the results of our current study.

VI of HCC tumor is considered to be a reflection of aggressiveness and has a well-known negative prognostic impact after hepatic resection [28]. However, little information is available regarding this tumor progression mechanism, which remains to be elucidated. A possible postulation is that portal vein or hepatic vein tumor invasion may simply be an effect of tumor topography, which means that this aggressive phenomenon may happen only because of the close anatomical proximity to neighboring vessels. A study comparing gene expressions between primary tumors and their paired portal vein tumor thrombi has demonstrated only a small difference [29]. However, studies focused on the mechanism of tumor metastasis have demonstrated the importance of phenotype changes in individual tumor cells [30, 31]. Recently, genomic studies have shown that unique genes and noncoding RNAs may have an important role in this mechanism [32, 33].

Serum levels of aspartate aminotransferase (AST) are one of the important prognostic factors after hepatic resection for HCC in this study. A study demonstrated that higher AST levels are positively correlated with an influx of hepatitis B virus [34]. In this study, 78.5% of patients have chronic B-viral hepatitis. Advancing underlying liver diseases may also be related to mitochondrial injury, which leads the release of AST [35]. So, elevated AST level may be indirectly reflecting the progress of hepatitis B. Multiple studies have supported that sustained viremia has a role in recurrence of hepatitis B virus-related HCC, and prevention effect of anti-viral therapy for recurrence [36].

The present study limitations include its retrospective nature and nonrandomized design, even though the data were prospectively collected. Furthermore, there was little information on important patient perioperative status, such as antiviral drug use, postoperative progression of underlying liver disease, or exposure to other carcinogens including alcohols, which have been considered to influence tumor recurrence or de novo malignancy. External validation of meaningful findings in this study is also needed in a multicenter-organized database setting. Unfortunately, there is a lack of clarity in the definition of McVI, leading to inter- and intra-pathologist variability in the evaluation of McVI in HCC [17]. However, all tumor tissues were evaluated by one liver-specialized pathologist with over 25 years of experience in this study. There is an attempt to establish a definition of McVI in HCC, using general histopathological principles, requiring prospective validation [37].

## Conclusion

McVI showed similar clinical significance compared with gross VI confined to segmental/sectional branches as a risk factor for tumor recurrence and poor survival of patients with HCC. Therefore, this study recommends considering McVI when estimating the tumor stage to predict the prognosis and to plan follow-up surveillance and additional treatment for patients with HCC.

## Abbreviations

AFP: α-fetoprotein
AJCC: American Joint Committee on Cancer
AST: Aspartate aminotransferase
BCLC: Barcelona Clinic for Liver Cancer
CI: Confidence interval
CT: Computed tomography
CTP: Child–Turcotte–Pugh
HCC: Hepatocellular carcinoma
HR: Hazard ratio
HVTT: Hepatic vein tumor thrombus
ICG-R15: Indocyanine green retention rate at 15 min
LCSGJ: Liver Cancer Study Group of Japan
LT: Liver transplantation
McVI: Microvascular invasion
OS: Overall survival
PVTT: Portal vein tumor thrombus
RFS: Recurrence-free survival
ROC: Receiver operating characteristic
TNM: Tumor node metastasis
UICC: International Union for Cancer Control
VI: Vascular invasion
